# Dig1 protects against cell death provoked by glyphosate-based herbicides in human liver cell lines

**DOI:** 10.1186/1745-6673-5-29

**Published:** 2010-10-27

**Authors:** Céline Gasnier, Nora Benachour, Emilie Clair, Carine Travert, Frédéric Langlois, Claire Laurant, Cécile Decroix-Laporte, Gilles-Eric Séralini

**Affiliations:** 1 Laboratory of Biochemistry EA2608, Institute of Biology, University of Caen, France; 2 CRIIGEN and Risk Pole MRSH, CNRS, University of Caen 14032, France; 3 Sevene Pharma, 30170 Monoblet, France

## Abstract

**Background:**

Worldwide used pesticides containing different adjuvants like Roundup formulations, which are glyphosate-based herbicides, can provoke some in vivo toxicity and in human cells. These pesticides are commonly found in the environment, surface waters and as food residues of Roundup tolerant genetically modified plants. In order to know their effects on cells from liver, a major detoxification organ, we have studied their mechanism of action and possible protection by precise medicinal plant extracts called Dig1.

**Methods:**

The cytotoxicity pathways of four formulations of glyphosate-based herbicides were studied using human hepatic cell lines HepG2 and Hep3B, known models to study xenobiotic effects. We monitored mitochondrial succinate dehydrogenase activity and caspases 3/7 for cell mortality and protection by Dig1, as well as cytochromes P450 1A1, 1A2, 3A4 and 2C9 and glutathione-S-transferase to approach the mechanism of actions.

**Results:**

All the four Roundup formulations provoke liver cell death, with adjuvants having stronger effects than glyphosate alone. Hep3B are 3-5 times more sensitive over 48 h. Caspases 3/7 are greatly activated in HepG2 by Roundup at non-cytotoxic levels, and some apoptosis induction by Roundup is possible together with necrosis. CYP3A4 is specifically enhanced by Roundup at doses 400 times less than used in agriculture (2%). CYP1A2 is increased to a lesser extent together with glutathione-S-transferase (GST) down-regulation. Dig 1, non cytotoxic and not inducing caspases by itself, is able to prevent Roundup-induced cell death in a time-dependant manner with an important efficiency of up to 89%, within 48 h. In addition, we evidenced that it prevents Caspases 3/7 activation and CYP3A4 enhancement, and not GST reduction, but in turn it slightly inhibited CYP2C9 when added before Roundup.

**Conclusion:**

Roundup is able to provoke intracellular disruption in hepatic cell lines at different levels, but a mixture of medicinal plant extracts Dig1 can protect to some extent human cell lines against this pollutants. All this system constitutes a tool for studying liver intoxication and detoxification.

## Background

Roundup (R) is the most widely used non-selective herbicide worldwide. It is comprised of a mixture of an isopropylamine salt of glyphosate (G) and adjuvants. G is considered as the active ingredient of R, although quantitatively it is a minor constituent, which is not supposed to be toxic alone in mammals [1]. Various adjuvants are present in R as secret of formulations [2], amplifying and thus allowing the G herbicide action, as well as its unintended toxic and endocrine disrupting effects in human placental cells [3]. The adjuvants, which are chosen from a long list that can vary from formulation to formulation [4], stabilize and help G penetration into cells. Among these are benzisothiazolone, isobutene, light aromatic petroleum distillate, methyl pyrrolidinone, polyethoxylated tallowamine or alkylamine (POEA), etc [2]. Some of these compounds may be genotoxic or form adducts with DNA [5]. It is thus important to compare different R formulations when studying this herbicide's toxicity. Moreover R residues are quite stable in rivers and soils [1]. G and its metabolite AMPA (aminomethyl phosphonic acid) are among the primary pollutants of surface waters (IFEN, 2006); they also enter the food chain [6]. These chemicals are found in the urine of agricultural workers [7]. The use of this herbicide is increasing as more than 75% of genetically modified edible plants have been designed to be used in conjunction with R. These plants are engineered to tolerate high intracellular levels of R [8]. We have also shown that the human embryonic kidney 293 cell line was even more sensitive to R, this was dose- and time-dependent [4]; and thus it was hypothesized that this could explain pregnancy outcomes and miscarriages reported for agricultural workers using G-based herbicides [9]. This is consistent with the fact that G-based herbicides have recently been shown to be endocrine disruptors in cell lines [10].

We know that xenobiotics have a main endpoint in the liver, which is the major detoxification organ. Here, we investigated the mechanism of action of R in the human liver cell lines available, HepG2 and Hep3B, which have been used as a model system to study xenobiotic toxicity, most prominently HepG2 [11,12]. We wanted to compare in the first instance the actions of four R formulations on both cell lines and then to detail the enzymatic pathways activated in HepG2.

Detoxifying mechanisms are frequently enhanced by plant extracts, which can provide additional protection against radicals and electrophilic compounds [13,14]. We have tested the ability of a new drug described for the first time, Dig1 (D), to protect cells from R intoxication. D contains plant extracts from *Taraxacum officinalis*, *Arctium lappa *and *Berberis vulgaris*. These herbal preparations were chosen in particular for their digestive detoxification or hepato-protective effects [15-20]. It was thus interesting to compare these general findings on plant extracts to some biochemically precise markers that could be modified in human hepatocytes, such as caspases 3/7, cytochromes P450, glutathione S-transferase (GST), and mitochondrial succinate dehydrogenase (SD), in order to detail the pathway(s) of action(s) of these mixtures used as medicinal plants *in vivo*, and thus to explore their cellular protective potential.

## Methods

### 1. Chemicals

Four main R formulations which have been used in agriculture (Monsanto, Anvers, Belgium) have been chosen in this study: Express^® ^7.2 g/l of G called glyphosate or N-(phosphonomethyl) glycine, product number 2010321; Bioforce^® ^360 g/l of G, product number 9800036; GT^® ^400 g/l of G, product number 8800425; GT+^® ^450 g/l of G, product number 2020448. The various herbicide formulations were prepared in Eagle's modified minimum essential medium (EMEM; Abcys, Paris, France), with 10% calf fetal serum from Cambrex (Verviers, Belgium) otherwise specified. G was from Sigma-Aldrich (Saint Quentin Fallavier, France), its called "2% solution" was equivalent in concentration to 2% R Bioforce^® ^and was prepared in serum free-medium, and adjusted to pH 5.8 of 2% R. D is a mixture of diluted plant extracts obtained by Sevene Pharma (Monoblet, France) from original independent macerates corresponding to 1/10 of dried plants in a water-alcohool solution of 45 to 55%. They are afterwards diluted in 70% alcohol with *Taraxacum officinalis *macerate at 10^-4^, *Arctium lappa *at 10^-4 ^and *Berberis vulgaris *at 10^-5^. D is prepared in the medium at 2% of the mixture in positive controls. The 3-(4,5-Dimethylthiazol-2-yl)-2,5-diphenyl tetrazolium bromide (MTT) and all other compounds, unless otherwise specified, were from Sigma-Aldrich. The MTT stock solution at 5 mg/ml in phosphate-buffered saline was diluted 10-fold in serum-free EMEM and then filtered through a 0.22 μm filter.

### 2. Cell cultures, Roundup and/or Dig1 exposures

**Figure 1:**
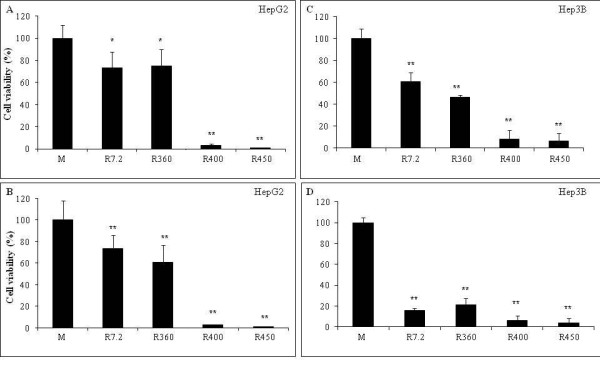
**Time-dependent effects of different Roundup formulations on HepG2 and Hep3B cell viability**. The formulations were applied during 24 h (A and C) or 48 h (B and D). These effects were evaluated by the MTT test (see Methods), measuring mitochondrial succinate dehydrogenase activity. The results are presented in percent compared to non treated cells (M). Cells were grown at 37°C (5% C0_2_, 95% air) in medium EMEM with 10% serum during 48 h to 80% confluence in 24-well plates, and then exposed to 4 different Roundup formulations. On × axis, concentrations of G in R in parenthesis: Express^® ^7.2 g/l of G, Bioforce^® ^360 g/l of G, GT^® ^400 g/l of G, GT+^® ^450 g/l of G, all at 0.5%. All experiments were repeated 3 times in triplicates. Statistically significant differences are calculated in comparison to control by a student *t*-test p < 0.01(**) and p < 0.05(*).

The hepatoma cell lines HepG2 and Hep3B were provided by ECACC, numbers 85011430 and 86062703, respectively. They are from Caucasian and Negroid hepatoma origins (from 15- and 8-year-old children respectively). Cells were grown in flasks of 75 cm^2 ^surface from Dutscher (Brumath, France) in phenol red-free EMEM containing 2 mM glutamine, 1% non-essential amino acid, 100 U/ml of antibiotics (mix of penicillin, streptomycin) and 10% fetal calf serum. For treatments, 50,000 cells were plated per well and grown at 37°C (5% C0_2_, 95% air) during a period of 48 h to 80% confluence in 48-well plates (except in Figure 1, 24-well plates were used). The cells were then exposed (24-72 h) to various concentrations of tested chemicals, which were replaced every 24 h for D studies. D was at 2%. For cytochromes and GST studies, before S9 fractions preparations, cells were treated in 25 ml and in 175 cm^2 ^flasks at 80% confluence. In this case after 24 h, another 25 ml was added as the second treatment. In all cases, medium M was used as control and R was present at the LC50, which was 25 ppm for R400 in these conditions, far below doses recommended in agriculture (1-2%, i.e. 10,000-20,000 ppm).

### 3. S9 fractions

The medium was removed, and cells dislodged by treatment with 7 ml of trypsin-EDTA (Lonza, France) and washed (PBS, Eurobio, France) twice by centrifugations (70 *g*, 5 min), at room temperature. Cells were then resuspended in 500 μl of 50 mM phosphate buffer pH 7.5 with 0.25 M sucrose, 1 mM DTT, homogenized and centrifuged at 9,000 *g*, 4°C for 30 min. The supernatants corresponding to the S9 fractions (membrane and cytosolic enzymes) were collected and frozen at -80°C until further evaluation for enzyme activities. Protein concentration was determined in each S9 fraction according to the Bicinchoninic Acid Protein Assay (Sigma, France).

### 4. Cell death measurement

The enzymatic MTT test is based on the cleavage of MTT into blue formazan by the mitochondrial enzyme succinate-dehydrogenase [21,22], it was used to evaluate human cell viability as described in our group [23]. The optical density was measured using a luminometer (Mithras LB 940, Berthold, France) at 570 nm. The crude protective actions were evaluated at the end of the treatment, by comparing the toxicity of R after treatment by D or not. As R toxicity is induced at the chosen LC50, the relative efficiency of the protective effect (recovering) is the percentage of recovered viability in the presence of D in comparison to the maximal toxic effect at LC50.

### 5. Caspase 3/7 activity measurement

The Caspase-Glo^® ^3/7 assay (Promega, Paris, France) in 96-well white plates (Dutscher, France) was a luminescent method designed for automated high-throughput screening of caspases activity, which is a measure of apoptosis. This method can measure caspase-3 and -7 activities in purified enzyme preparations or cultures of adherent or suspended cells [24-26]. The assay provides a pro-luminescent caspase-3/7 substrate, which contains the tetrapeptide sequence DEVD. This substrate is cleaved to release amino-luciferin, a substrate for luciferase, and the production of light is proportional to the quantity of amino-luciferin released and therefore proportional to caspase. The Caspase-Glo^® ^3/7 reagent has been optimized for caspase activity, luciferase activity and cell lysis. The addition of the single Caspase-Glo^® ^3/7 reagent, in an "add-mix-measure" format, results in cell lysis followed by caspase cleavage of the substrate and generation of a "glow-type" luminescent signal. After cell cultures were exposed to 50 μL of various dilutions, an equal volume of Caspase-Glo^® ^3/7 reagent was added to each well. Plates were then agitated 15 min and incubated 45 min at room temperature in the dark, to stabilize the signal before measuring luminescence. The negative control was the serum-free medium, the positive control was the active Caspase-Glo^® ^3/7 reagent mixed with cells treated only with serum-free medium to determine the basal activity of the caspases 3/7. Luminescence was measured using the luminometer Mithras LB 940 (Berthold, Thoiry, France) at 565 nm.

### 6. Cytochrome P450 activity measurement

The cytochrome P450 3A4, 2C9, 1A2 and 1A1 activities were quantified by the P450 Glo™ Assays (Promega, France), as described by Yueh *et al*. [27]. Each Cytochrome P450/1 M KPO_4_/Substrate Reaction mixture containing the S9 fractions (duplicate) was pre-incubated at 37°C for 10 min in white 96-well plates (Dutscher, France). The Cytochrome P450 CYP1A1 Reaction mixture contained 135 μg of the human liver S9 fraction as control (Tebu-Bio, France) or cell S9 fraction with 60 μM luciferin-conjugated substrate (luciferin-6'-chloroethyl ether) and 200 mM KPO_4 _buffer in a final volume of 25 μl. For CYP1A2 130 μg S9 fractions were used with 200 μM substrate of luciferin-6'-methylether; for CYP2C9 160 μg S9 and 200 μM substrate of 6'-deoxy luciferin but with 50 mM KPO_4 _buffer in 25 μl. CYP3A4 was measured with 170 μg S9 with 100 μM substrate of luciferin-6'-benzylether but with 400 mM KPO_4 _in 25 μl. The enzymatic reaction was initiated by adding 25 μl of NADPH regenerating system to each well. It contained 2.6 mM NADP^+^, 6.6 mM glucose-6-phosphate, 0.8 U/ml glucose-6-phosphate dehydrogenase and 6.6 mM MgCl_2_.

The plate was then incubated at 37°C for 20 min for CYP1A1 and CYP1A2, and, for 30 min for CYP2C9 and CYP3A4. The reconstituted Luciferin Detection reagent (50 μl) was added before mixing for 10 s and incubating at room temperature for 90 min in order to stabilize the luminescent signal. The luminescence was then read with a luminometer (Veritas Turner Biosystems). Three independent experiments were carried out using three independent batches of S9 fractions.

### 7. GST activity measurement

The protocol was adapted from Habig *et al*. [28]. Briefly, 320 μg (50 μl) of the human liver S9 fraction (positive control) or cell S9 fraction was mixed with 10 μl of 100 mM GSH and 930 μl phosphate buffer in duplicate. Reduced L-glutathione (GSH) was dissolved in deionized water; pH 6.5 buffer was prepared by mixing 0.7 volume of 0.1 M KH_2_PO_4 _and 0.3 volume of 0.1 M Na_2_HPO_4_. The reaction was initiated by 10 μl of 100 mM 1-chloro-2,4-dinitrobenzene (CDNB) substrate. The CDNB was dissolved in 95% ethanol at a concentration of 100 mM (20.3 mg/ml). After a 90 s incubation at 37°C, the optical density was measured at 340 nm every 30 s for 90 s with a SmartSpec 3000 Spectrophotometer (Bio-Rad, France). Three independent experiments were carried out using three independent batches of S9 fraction.

### 8. Statistical analysis

The experiments were repeated 3 times in different weeks in triplicate (n = 9) unless otherwise specified. All data are presented as the mean ± standard error (S.E.M.). Statistical differences were determined by a Student unpaired *t*-test using significant levels of p < 0.01 (**) and p < 0.05 (*).

## Results

**Figure 2:**
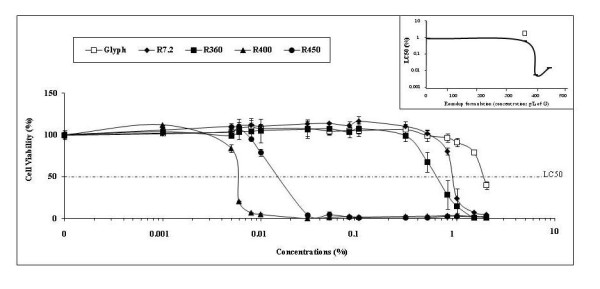
**Dose-dependent effects of Glyphosate and different Roundup formulations on HepG2 viability**. The formulations were applied during 24 h without serum (even for G) in 48-well plates, after reaching 80% confluence with serum-containing medium. These effects and the formulations with G concentrations (7.2 to 450 g/l) indicated with symbols were evaluated as described in Fig. 1. All experiments were repeated 4 times in triplicates. The curve in frame summarizes the nonlinear dose effects of R formulations on HepG2. The LC50 (%) values are compared for the 4 R and G (in similar conditions) as a function of G concentrations in the formulations. The LC50 for G alone is indicated by the empty square above the curve.

Figure 1 presents the different time-dependent effects of various R formulations at 0.5% on viability of liver cell lines HepG2 and Hep3B. The R formulations contained different concentrations of both G and various adjuvants. Both cell lines showed approximately similar growth rates for around 32 h in control medium (M). In both cell lines, growth rate was easily disrupted by any R formulation, but different R formulations had different effects. In the case of R7.2 and R360, Hep3B cells were approximately 3-5 times more sensitive than HepG2 over 48 h. However, in the case of R400 and R450 at 0.5% the two cell lines were roughly equal in sensitivity. These two R formulations were found to be most rapid-acting and toxic. Based on this observation these were chosen for use in subsequent experiments. Cell death was estimated by inhibition of succinate-dehydrogenase and thus of mitochondrial metabolism. In both cell lines, mortality increases with G concentrations and time of exposure to all 4 R formulations, however the increase is not proportional to G concentration (insert Figure 2). The first two formulations demonstrate similar toxicities despite having quite different concentrations of G (7.2 and 360 g/l of G, respectively), along with adjuvants; the two other formulations show higher toxicity as previously explained. This dose-dependent effect is clearly illustrated in Figure 2 with the two groups of decreasing curves with the two families of R (R400 and R450 on one side, first toxic family, and R7.2 and R360 on the other). It also becomes obvious that G has no toxic action alone under the conditions used in this study (empty squares, Figure 2).

**Figure 3:**
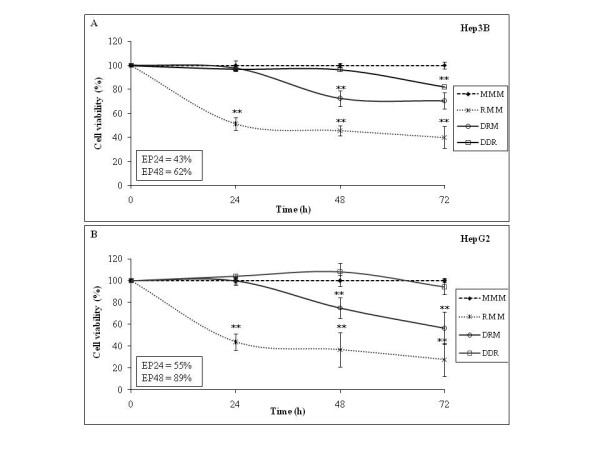
**Dig1 general preventive effect of R400 toxicity on Hep3B and HepG2 during 72 h**. In frames on the right, each letter (M, R or D) indicates 24 h of successive cell exposures to the corresponding conditions (Medium alone, Roundup, Dig1). The results were evaluated as in Fig. (**1**). To measure preventive effects, D at 2% was applied during 24 or 48 h before R (400 g/l at LC50 in these conditions, 40 ppm for Hep3B and 96 ppm for HepG2): corresponding treatments called DRM or DDR. R was applied alone as negative control during 24 or 72 h (RMM, RRR), or before D to assess curative effects (RDM, RMD or RDD), all these curves are superimposed, and thus only RMM is shown as negative control. No curative effect is evidenced in these conditions. Efficiencies of protection (EP) after 24 or 48 h of treatment by D are indicated in frames.

We identified the LC50 of R400 (GT^®^) in 24 h in 48-well plates as being 40 ppm for Hep3B and 96 ppm for HepG2. No difference was seen between HepG2 and Hep3B cells in their sensitivity to R400 when exposed at relatively high concentrations in Figure 1. Titrations to determine the LC50 of R400 revealed clearly that Hep3B cells were more sensitive to R400 than were HepG2. We then tested the impact of 2% D at these conditions of R intoxication (Figure 3). We confirmed R400 toxicity to hepatocyte-derived cell lines exposed at the LC50 for 24 h, and found that D was able to prevent this toxicity. First, we demonstrated that D alone was not toxic, at 2% for as long as 72 h, nor was it able to inhibit mitochondrial metabolism (data not shown). Then we observed that pre-treatment with D (in comparison to control M) had a time-amplified protective effect from the most toxic R. After 24 h of D exposure and 24 h of R, the efficiency of protective action of D reached 43% for Hep3B, and 55% for HepG2. After 48 h of D and 24 h of R, the efficiency of D reached 62% and 89% respectively. These effects were proportional to time (compare curve DRM to RMM). No curative effect (positive D action after R) was observed under these conditions with these cells since post-treatment with D did not influence R toxicity (curves are superimposed with RMM, see Figure 3, legend).

**Figure 4:**
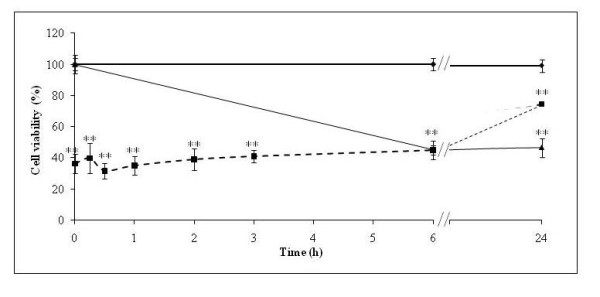
**Time necessary for Dig1 pre-incubation to achieve a significant preventive effect of subsequent R intoxication**. The study is performed with R400 intoxication on HepG2, cell viability is measured as in Fig. (**1**). First D (2% - dotted line with squares) was applied (time on × axis) 15, 30 min, 1 to 24 h before R400 (96 ppm during 24 h - grey line with triangles), in comparison to M (black line with diamonds). More than 40% viability (R effect in these conditions) is obtained only after at least 6 h of D exposure (increasing dotted line).

**Figure 5:**
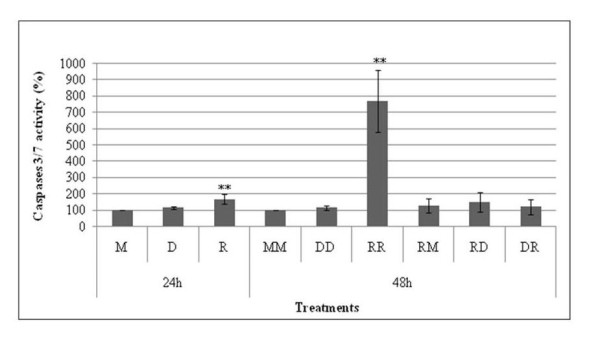
**Roundup and Dig1 on HepG2 caspase 3/7 activity**. These effects were evaluated by the caspases Glo^® ^3/7 assay, the results are presented in percent compared to untreated cells (M). Cells were grown at 37°C (5% C0_2_, 95% air) in serum containing medium during 48 h to 80% confluence in 96-well plates, and then exposed to different treatments (R450: 60 ppm, D 2%) without serum. The formulation was applied during 24 h (M, D, R) or 48 h (MM to DR).

The mechanism was studied in more detail in HepG2 where the enzymes are better characterized and assessed. First, we evaluated the time-course for onset of the preventive effect of D, by incubating cells with D and removing it before R addition (Figure 4). We show that the efficiency of protection of D is established (53% in this case) at 24 h, but can be observed as early as 6 h after adding D to cells. At this time, protection from the toxicity of R was significant. We then investigated what might be the metabolic target of the protective effect of D. Caspases 3/7 are shown in Figure 5 to be activated up to 156% by 24 h exposure to R and up to 765% by 48 h exposure (comparison was to the control M at 24 h and MM at 48 h). After 24 h of exposure, if R is replaced by M, the caspases recover in 24 h to their initial activity. Figure 5 shows that D does not induce caspases itself, but appears to prevent induction of caspases by R (DR). Considering caspases activities as an early sign of apoptosis, these results confirm lack of D toxicity and the ability of D to protect from R toxicity.

**Figure 6:**
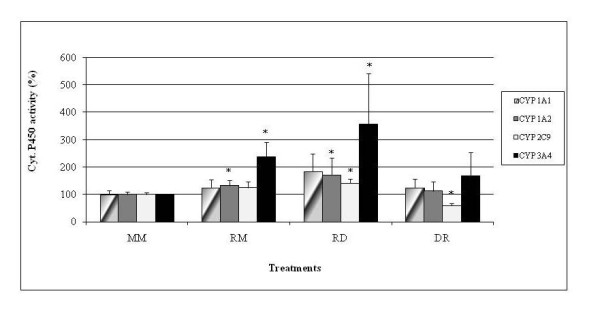
**Roundup and Dig1 on HepG2 Cytochromes P450 activities**. These effects were evaluated by the P450 Glo^® ^assay, the results are presented as percent relative to control (MM). Cells were treated before S9 fractions preparations in 25 ml and in 175 cm^2^-flasks at 80% confluence. After 24 h of M, R (LC50 of R400, 25 ppm in these conditions) or D (2%), another 25 ml was added as the second treatment.

**Figure 7:**
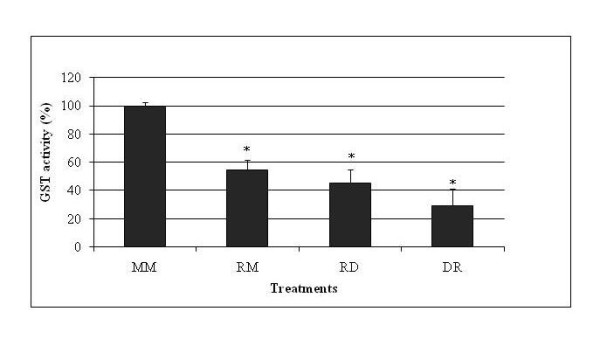
**Roundup and Dig1 on HepG2 glutathione-S-transferase activity**. The results are presented in percent compared to untreated cells (MM). Cells were treated before S9 fractions preparations in 25 ml and in 175 cm^2^-flasks at 80% confluence. After 24 h of M, R (LC50 of R400, 25 ppm in these conditions) or D (2%), another 25 ml was added as the second treatment.

**Figure 8:**
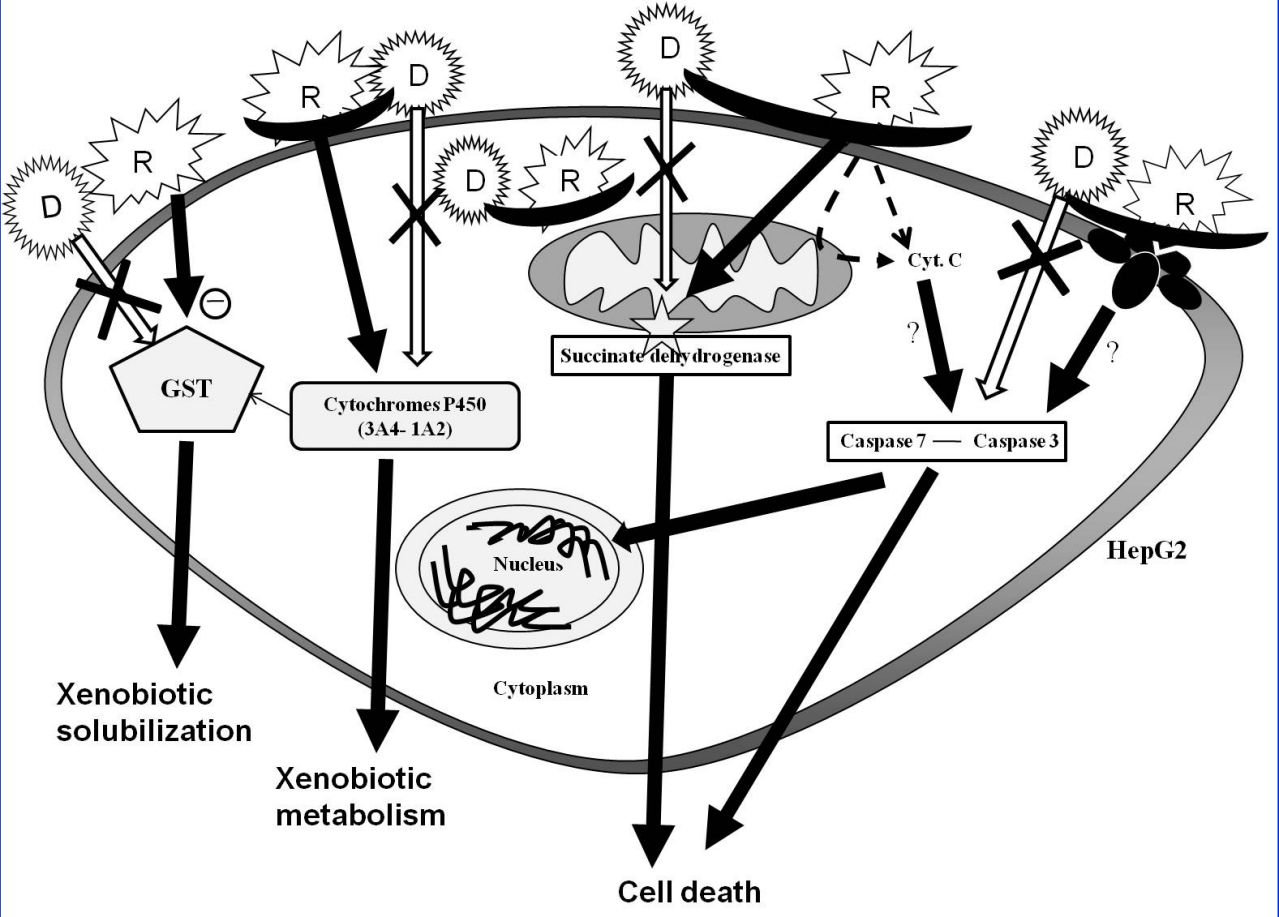
**Roundup (R) mechanisms of action and prevention by Dig1 (D) in human hepatocytes HepG2**. The different pathways of action identified in this research are summarized with black arrows: action via on mitochondrial succinate dehydrogenase, and action via caspases 3/7 inducing cell death (directly or indirectly through cytochrome C, and possibly via death cell receptors), action via cytochromes CYP3A4 and CYP1A2 stimulating the formation of metabolites, and finally action via the inhibition of Glutathione-S-transferase (GST) blocking metabolites derivatization and excretion. D does not act itself at these levels (crossed empty arrows) but prevents R toxicity (black thick lines).

In addition to caspases, we examined the effects of R on cytochromes, finding that R does not activate all cytochromes but is able to enhance more specifically CYP3A4 (to 240-360%) and to a lesser extent CYP1A2 (to 130-170%, Figure 6). D does not enhance these cytochromes by itself (RD versus RM, Figure 6); but it weakly increases CYP2C9 in combination with R (to 140%), when added after it, even though this is not statistically different from RM treatment (but from control M alone). Once again, based on this additional parameter, D confirmed its ability to block R toxicity: if D is applied before R no cytochrome activity was stimulated, CYP2C9 was even weakly inhibited (40%). By contrast, in Figure 7, it is shown that R inhibits GST almost by half, and D does not modify this effect either before or after R treatment. Figure 8 summarizes the results obtained on the different pathways of R and D actions on HepG2.

## Discussion

This work evidences for the first time the effects of several formulations of the major herbicide worldwide (R) on human hepatic cell lines available, which are widely recognized as models to study xenobiotic actions. We tested R at sub-agricultural levels; the LC50 measured in this work is 10 times for Hep3B and 4 times for HepG2 below the maximum level of residues authorized in some feed (400 ppm, [29]). We found that the cell sensitivity depended on the nature of the R formulation. Both cell lines have retained the activities of the drug metabolism phase I and phase II enzymes involved in activation and detoxification of genotoxic carcinogens [30,31]. HepG2 cells are in general considered a better model since they have three-fold higher levels of CYP1A1 and glutathione-S-transferase (GST) than Hep3B [32]. For instance, it was observed that HepG2 are more sensitive than Hep3B to cisplatin [33], to dietary genotoxins [32] and to genotoxicants [12]. In our hands it was the opposite for R, overall in 48 h. In fact, both cell lines are from different genetic origins, from different boys at different ages, and thus have specific enzymatic equipments including cytochromes P450. Greater sensitivity was observed with Hep3B. It was thus important to obtain results in both lines that confirmed R toxicity in all human models tested up to now, including embryonic 293 cells, umbilical cord cells, placental derived JEG3 cells and microsomes from fresh placenta [3,4,34]. It was evidenced that cell death increased with time of exposure for all R formulations, this phenomenon shows that the threshold of toxicity depends not only on the dose but also on time, as previously clearly demonstrated on embryonic and placental cells [4]. It was also shown that toxicity was dependent on the nature of the adjuvants present in different R formulations. Moreover, the action of serum only temporarily buffered R toxicity. We found that serum-free culture medium revealed essentially the same xenobiotic impacts that are visible 1-2 days later in serum. Thus, although the timecourse of action is delayed, the pathways of actions appear similar [4].

Indeed, the G cytotoxic effects do not vary linearly with dose; this demonstrates the differential roles of adjuvants in the amplification of toxicity, since G has no toxicity alone at these concentrations. The adjuvants added to G in various R formulations are considered manufacturing secrets, but obviously do not form an inert part of the composition. One mechanism of adjuvant action is most probably to form detergent vesicles that allow cell membrane opening and penetration of G, and that most probably facilitate bioaccumulation of G, metabolites and adjuvants, and gene disruptive effects, which could explain time-amplified effects. A very small quantity of adjuvants combined with G has been already demonstrated to have similar effects to R [3]. It is a recognized fact that mixtures of xenobiotics have synergistic effects [35]. We observed that whatever the nature of the various adjuvants is in the 4 R formulations, the mechanism of toxicity is similar on several crucial endpoints: namely SD, AK, Caspases 3/7 [34]. Only the threshold of toxicity is different.

In this study, we sought to understand the mechanism of toxicity of the two R formulations that have the most rapid toxic effects on hepatic cells. We also evaluated whether it was possible to prevent R toxicity by D. D is a newly described product comprised of a mixture of extracts from *Taraxacum officinalis*, *Arctium lappa *and *Berberis vulgaris*. *Taraxacum *was cited for protective effects in the digestive system [19,20], also anti-tumoral [36] and anti-oxydant effects [37]. *Arctium lappa *is also found to be hepato-protective [17,18], as well as *Berberis *[16].

First of all, the lack of D impact on cell viability in comparison to controls indicates that it is not cytotoxic at 2%. It was hypothesized first that either D does not penetrate cells without embedding cell-cell interactions, or it is relatively inert on several important markers of cell function at this concentration. Our results are consistent with D penetrating the cells and not just forming a shield that prevents R from penetrating. This is verified since effective protection by D necessitates more than 6 h of contact, and because D modifies enzymatic activities, as detailed below. As a matter of fact, D shows a clear and very strong efficiency of protective action against the toxicity of R in both hepatic cell lines; protection is up to 89% for HepG2 in 48 h. It is not completely excluded that this important action of D can occur by preventing entrance of R into cells, normally helped by adjuvants. However D clearly has intracellular actions, including prevention of caspases 3/7 activation and CYP3A4 enhancement, both provoked by R. CYP 3A4 was induced by Roundup in comparative manner and in similar time than with other xenobiotics Bisphenol A, DDT and phtalates, respectively [38-40].

Other metabolic/biochemical effects of D components have also been reported, for instance, the protective effects on acute pancreatitis in rats [20] through IL-6 reduction, as well as the suppression of reactive oxygen species, nitric oxide and lipid oxidation [37]. Polyphenolic compounds present in D are considered hepatoprotective [42-44] but the time-dependent protective effects on the crucial mitochondrial succinate dehydrogenase and caspases 3 and 7 had not been demonstrated before.

Since HepG2 are more resistant and D more preventive on this line, we decided to detail the signalling pathway of R and D actions in these cells. Moreover, the caspases, cytochromes and GST activities are more fully documented in this hepatic cell line [45-47]. The measurement of caspase 3/7 activity has been recognized as a marker for apoptosis in HepG2 cells [48].

Caspases 3/7 have been rarely measured in HepG2, and found to be induced 200% by 30 μM Tamoxifen in 24 h [48]. Their induction by R in our study (156% in 24 h and 765% in 48 h) confirms the proposed apoptotic mechanism activated by R. Of course, this does not exclude some necrosis as a primary mode of cell death as we previously showed [34]. Our results also show that D alone does not interfere with caspases but may possibly prevent R impact on these enzymes.

At lower levels and even with the buffering effect of serum already documented [4], D also prevents CYP3A4 induction: CYP3A4 is by far the major target of R among the phase I activating enzymes measured. CYP3A4 is the most abundant P450 expressed (60%) in human liver [49]. It is involved in the bioactivation of environmental procarcinogens, such as aflatoxin B1 [50] and benzo[a]pyrene among a number of others [51]. Some organochlorine pesticides can activate the PXR, a nuclear hormone receptor [52] that regulates CYP3A4 gene transcription in particular in liver. R components may also induce this process. The results with CYP1A2 (slight induction by R) show that its action does not depend on a unique pathway, like the results on CYP2C9. Only a few reports are available on CYP2C9, which is inhibited by extracts of other plants such as pineapple [53].

Similarly, only some xenobiotics such as polychlorobyphenyls inhibit GST; this is typical of estrogen-like effects [54] already observed with R on aromatase [3,4]. The disruption of xenobiotic metabolism by R in our study, which increased some cytochromes P450 and decreased GST, may alter liver detoxification function, leading to the accumulation of toxic reactive oxydated molecules [54] in a manner that could be prevented by D in this study. In fact, GST inhibition, if R intoxication continues, could lead to the accumulation of reactive compounds, due to disruption of xenobiotic excretion.

In conclusion, it is demonstrated and explained that high hepatic cell line mortality is provoked by R, at doses far below those used in agriculture. The mechanism of action on various essential enzymes is detailed in Figure 8. This impact on cell death was observed at doses far smaller than legally allowed residues of G in GM food or feed (400 ppm, [29]), in our study LC50 was in comparison 40 to 96 ppm. Of course G can be metabolized and excreted out of the body but this has to be balanced in regard to its cell penetration and bioaccumulation due to adjuvants. In these conditions, this can be almost totally prevented in HepG2 in 48 h by a specific drug, D, that mostly prevents caspases 3/7 activation and CYP3A4 enhancement, both provoked by R. However it does not prevent GST inhibition by R that could lead to accumulation of toxic reactive compounds. Since the use of cell lines allows longer experiments, and since cell lines may in some instances be less sensitive to xenobiotics than primary cultures [55], this system with R and Dig1 constitutes an interesting tool for studying liver intoxication and detoxification. The activity of Dig1 should be now also tested *in vivo *in animal experiments.

## Competing interests

The authors declare that they have no competing interests. The development of Dig1 in Sevene Pharma was performed completely independently of its assessment. The scientists in the University of Caen in charge of the assessment of xenobiotics or plant extracts declare no financial or other interests in the development of these products.

## Authors' contributions

CG carried out the cellular, biochemical and molecular studies, participated in drafting the manuscript. NB and EC reproduced and helped the cellular experiments. CT participated in the methodological and protocol advices, and discussions. FL initiated the collaboration in Sevene Pharma and carefully followed the first sets of experiments for the protocol design. CL participated in Dig1 conception and discussions. CDL directed Dig1 assessment for Sevene Pharma and GES conceived the study, the final version of the manuscript, participated in the design of the work and was responsible for the coordination. All authors read and approved the final manuscript.
